# Tick Bite With Target Lesion Complicating Routine Cast Immobilization

**Published:** 2015-04-17

**Authors:** Tyler Snoap, Todd Ruiter, Benedict Thomas Harter

**Affiliations:** ^a^Department of Orthopedics, Western Michigan University Homer Stryker M.D. School of Medicine, Kalamazoo; ^b^Borgess Medical Center, Kalamazoo, Mi; ^c^Robley Rex Veterans Administration Medical Center, Louisville, Ky

**Keywords:** cast care, tick-borne disease, target lesion, Lyme disease, erythema migrans

**Figure:**
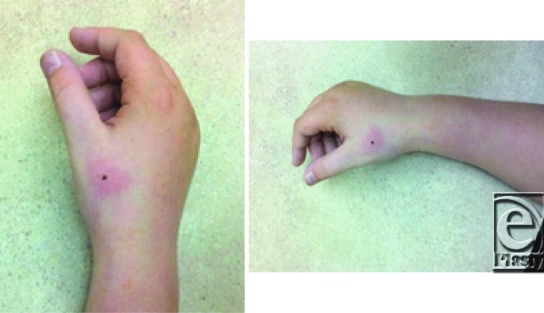


## CASE DESCRIPTION

An 8-year-old boy in southwest Michigan was found to have a tick and target lesion after routine cast care for a first metacarpal fracture. Serological markers were negative for Lyme disease; however, oral doxycycline was prescribed for 3 weeks. Final follow-up did not reveal musculoskeletal or infectious morbidity.

## QUESTIONS

1. **How are ticks vectors of disease transmission?**
2. **What are the endemic areas for tick-borne pathogens?**
3. **What is Lyme disease and how is it treated?**
4. **Can physicians take steps to prevent tick-borne illnesses while immobilizing patients?**

## DISCUSSION

Ticks are hematogenous invertebrates with a life cycle that is unique, making them capable carriers of other organisms, some of which can lead to human disease. Most commonly, but not exclusively, ticks spread pathogens to hosts through salivary secretions; these pathogens can be viruses, bacteria, fungi, protozoa, or helminthes.^1^ Ticks are second only to mosquitoes as carrying vectors for human disease.^2^ Anaplasmosis, babesiosis, ehrlichiosis, Rocky Mountain spotted fever, tularemia, and Lyme disease are examples of conditions that can result from tick vectors carrying pathogens. More than 90% of all vector-borne disease cases in the Unites States are due to Lyme disease, caused by the spirochete *Borrelia burgdorferi sensu lato*.^3^

Specific tick subtypes have a geographic predilection. For example, the American dog tick (*Dermacentor variabilis*) is most commonly associated with transmitting Rocky Mountain spotted fever as well as tularemia. This is most commonly seen in the Eastern United States as well as some areas of California. The blacklegged tick (*Ixodes scapularis*) is associated with transmitting anaplasmosis, babesiosis, and Lyme disease. This tick's endemic region is the Midwest and Eastern border of the United States.^4^

Physicians practicing in these endemic regions must be cognizant of potential infectious disease hazards ticks pose in terms of disease transmission. Prompt diagnosis and treatment are imperative. Failed recognition of a tick bite with associated cutaneous lesion erythema migrans may result in Lyme disease, which can have devastating consequences on the human host. This disease can wreak havoc on the cardiac, neurological, and musculoskeletal systems, amongst others. Timely recognition via serological testing with ELISA (enzyme-linked immunosorbent assay) and confirmation with Western blot is paramount to diagnosis. Treatment of Lyme disease is antibiotics with varying dosages and forms based on specific organ system involvement and phase of the disease.^5^

Preventative measures for tick-borne illnesses focus on decreasing exposure, detection, and early removal of ticks once detected. Frequent tick checks in endemic areas, light-colored clothing for early identification, and layered clothing to prevent tick attachment are all encouraged. These strategies are difficult to implement in a patient treated with cast or splint immobilization. A 1998 *New England Journal of Medicine* report reviewed early success of vaccination as a means of prevention of Lyme disease.^6^ However, potential pitfalls including autoimmune arthritis, cost, and need for repeated vaccinations have hindered further research into broader Lyme disease vaccination. Pesticides such as acaricides are used in agricultural and residential applications to controlling tick populations.^7^ We are unable to find any published work exploring pesticide application in cast or splint materials to prevent tick bites. The toxicity of pesticides varies by chemical and dose, with potential side effects ranging from mild skin irritation to central neurotoxicity.^8^ As incidence and recognition of Lyme disease continue to rise, these may be viable areas for further research.

Cast immobilization is a staple of hand surgery. We are unable to find another reported case of tick bite with associated target erythema as a complication of cast immobilization. Preventing the tick bite is key toward preventing infectious disease transmission. There may be a role for further investigation of pesticide incorporation into casting or splinting materials when treating high-risk populations in endemic areas.
